# The impacts of self-expectation leadership and organizational commitment on hand hygiene behavior of medical staff based on the theory of implicit leadership

**DOI:** 10.3389/fpsyg.2022.992920

**Published:** 2022-11-14

**Authors:** Qianning Wang, Xiaoquan Lai, Feiyang Zheng, Tiantian Yu, Lu Wang, Yuanyang Wu, Kang Wang, Xinping Zhang, Qian Zhou, Li Tan

**Affiliations:** ^1^School of Medicine and Health Management, Tongji Medical College, Huazhong University of Science and Technology, Wuhan, China; ^2^Department of Nosocomial Infection, Tongji Hospital, Tongji Medical College, Huazhong University of Science and Technology, Wuhan, China; ^3^School of Nursing, The Hong Kong Polytechnic University, Hong Kong, Hong Kong SAR, China; ^4^Department of Hospital Infection Management, Wuhan Children’s Hospital, Wuhan Maternal and Child Healthcare Hospital, Tongji Medical College, Huazhong University of Science and Technology, Wuhan, China

**Keywords:** positive traits of self-expectation leadership, negative traits of self-expectation leadership, organizational commitment, hand hygiene behavior, medical staff, implicit leadership theory, impact mechanism, healthcare-associated infection

## Abstract

Hand hygiene behavior (HHB) in healthcare settings remains suboptimal globally. Self-expectation leadership and organizational commitment are emphasized as important factors influencing HHB. However, there are no studies to support any relationship between self-expectation leadership and organizational commitment to HHB. This study will fill the gap by applying implicit leadership theory (ILT) to support the further promote HHB among medical staff. A cross-sectional study of 23,426 medical staff was conducted in all second-level and third-level hospitals in Hubei province, China. Based on ILT, an online self-administered and anonymous questionnaire was designed for measuring the medical staff’s self-expectation leadership, organizational commitment, and HHB based on Offermann’s 8 dimensions scale, Chang’s 3 dimensions scale, and the *specification of hand hygiene for healthcare workers*, respectively, in which self-expectation leadership was divided into positive traits and negative traits parts. The structural equation model was used to examine the direct, indirect, and mediating effects of the variables. Positive traits of self-expectation leadership had a positive effect on organizational commitment (β = 0.617, *p* < 0.001) and HHB (β = 0.180, *p* < 0.001). Negative traits of self-expectation leadership had a negative effect on organizational commitment (β = –0.032, *p* < 0.001), while a positive effect on HHB (β = 0.048, *p* < 0.001). The organizational commitment had a positive effect on HHB (β = 0.419, *p* < 0.001). The mediating effect of the organizational commitment showed positively between positive traits of self-expectation leadership and HHB (β = 0.259, *p* < 0.001), while negatively between negative traits of self-expectation leadership and HHB (β = –0.013, *p* < 0.001). Positive traits of self-expectation leadership are important predictors of promoting organizational commitment and HHB, while negative traits of self-expectation leadership have a limited impact on organizational commitment and HHB in the field of healthcare-associated infection prevention and control. These findings suggest the need to focus on positive traits of self-expectation leadership; although negative traits of self-expectation leadership can also promote HHB to a lesser degree among medical staff, it will reduce their organizational commitment.

## Introduction

Hand hygiene behavior (HHB) is an important element of healthcare-associated infection (HAI) prevention and control (de Kraker et al., 2022). HAI is one of the most common types of adverse events affecting hospitalized patients (Brennan et al., 2004; Desikan et al., 2005; Garrouste-Orgeas et al., 2012). It was estimated that more than 91,000 and 99,000 people died as a direct result of HAI each year in Europe and the United States, respectively (Cassini et al., 2016; Magill et al., 2018). However, HHB in healthcare settings remains suboptimal globally (Lotfinejad et al., 2021), especially in developing countries (Assefa et al., 2021). It was reported that the HHB level was 9% in low-income countries, and rarely exceeded 70% in high-income countries (Erasmus et al., 2010; Lambe et al., 2019).

Several studies have pointed out barriers to low HHB levels including high workload, understaffing, lack of time and facilities, ineffective education, lack of role models, inadequate safety culture, forgetting and concerns about dry or cracked skin, and so on (ISHN, 2017; Sadule-Rios and Aguilera, 2017; Birnbach et al., 2019). Promotion measures include daily audits, monthly staff education, quarterly workshops, posters and reminders in strategic places in the wards (Bukhari et al., 2011; Lytsy et al., 2016; Soboksa et al., 2021). Besides, leadership and organizational commitment are emphasized as important factors influencing HHB in recent years (Boscart et al., 2012; Tan and Olivo, 2015; Linam et al., 2017), especially self-expectation leadership (Lieber et al., 2014; Pereira and Stornelli, 2022). Improving medical staff’s self-expectation leadership can increase HHB compliance by twofold and this increase was sustained over a 20-month follow-up period (Aboumatar et al., 2012). However, there are no studies to support any relationship between self-expectation leadership and organizational commitment to HHB.

Some policies have highlighted the importance of self-expectation leadership and organizational commitment to improving health (European Centre for Disease Prevention and Control, 2008; OEHSS, 2012; PSNet, 2019; WHO, 2020). The Office of Environment, Health, Safety & Security encouraged medical staff to strengthen self-expectation leadership to sustain a high-performing organization (OEHSS, 2012). World Health Organization issued the *State of the world’s nursing 2020: investing in education, jobs, and leadership* to advocate that strengthening medical staff’s leadership was an inevitable trend (WHO, 2020). Europe’s Center for Disease Control and Prevention pointed out that organizational commitment was a key role in promoting HAI prevention and control levels (European Centre for Disease Prevention and Control, 2008). Department of Health & Human Services in the U.S. also claimed the importance of organizational commitment to establishing a culture of safety (PSNet, 2019).

### Theoretical framework

To explore the relationship among medical staff’s self-expectation leadership, organizational commitment, and HHB, the implicit leadership theory (ILT) is considered appropriate (Eden and Leviatan, 1975). ILT was first proposed by Eden and Leviatan based on the notion of implicit theories of personality (Eden and Leviatan, 1975) and then used for research in the field of education and business administration (House et al., 2002; Sharifirad and Hajhoseiny, 2018). Sharifirad and Hajhoseiny (2018) researched the mechanism of teachers’ expectation leadership, cognition, and behavior based on the ILT. House et al. (2002) launched a global survey of middle managers in food processing, finance, and telecommunications industries based on the ILT. However, little is known about applying the theory in the medical field.

The framework of ILT assumed that expectation leadership can influence an individual’s cognition (organizational commitment) and then influence behavior (HHB) (Anderson, 1966; Eden and Leviatan, 1975; Lord and Maher, 1993). The main body of hand hygiene behavior was medical staff, and many researchers have pointed out that expectations of themselves were key to improving cognitive and behavioral levels (Bandura, 1977). Thus, in this study, we extended expectation leadership to self-expectation leadership to better validate its effectiveness in the field of HAI prevention and control (Manz and Sims, 1986, Manz and Sims, 1987; Chou, 2002). Furthermore, different types of leadership traits resulted in different effects on cognition and behavior (Lord et al., 1984; Kirkpatick and Locke, 1991). Therefore, in this study, we also divided self-expectation leadership into two parts: positive traits of self-expectation leadership and negative traits of self-expectation leadership to explore self-expectation leadership systematically (Chen, 2011).

In this study, positive traits of self-expectation leadership referred to the positive leadership level that medical staff expects of their own, such as morality (Neuhaus, 2020). Negative traits of self-expectation leadership referred to the negative leadership level that medical staff expects of their own, such as tyranny (Hughes et al., 2002). Organizational commitment refers to a common cognition that medical staff trust in the goals and values of the respective organization (Becker, 1960), including value commitment, effort commitment, and retention commitment (Chen et al., 2017). HHB referred to medical staff sanitizing hands with an alcohol-based hand rub under some specific situations (WHO, 2009), such as before and after interacting with patients (Landers et al., 2012).

To sum up, positive and negative traits of self-expectation leadership were linked with organizational commitment and then HHB.

### Literature review

Literature showed that positive traits of self-expectation leadership positively influenced organizational commitment and negative traits of self-expectation leadership negatively influenced organizational commitment in the oil industry (Chen, 2011). Studies in the field of teaching have also confirmed the conclusion (Çayak, 2021).

Many works of the literature showed that organizational commitment positively influenced HHB (Boscart et al., 2012; Tan and Olivo, 2015). A study pointed out that factors such as staff commitment of the department heads were perceived to be significant in promoting hand hygiene practices (Tan and Olivo, 2015). Another study intervened in nurses’ organizational commitment, and the intervention group showed better hand hygiene practices (Boscart et al., 2012).

The results of the literature review showed that both positive and negative traits of self-expectation leadership positively influenced HHB or other healthy behavior (Lieber et al., 2014; Pereira and Stornelli, 2022). A study pointed out that positive traits of self-expectation leadership improvement may play a key role in sustaining hand hygiene adherence (Lieber et al., 2014). Another study showed that negative traits of self-expectation leadership can improve epidemic control levels significantly (Pereira and Stornelli, 2022).

Although there were no studies on self-expectation leadership and organizational commitment to HHB, some literature showed that positive traits of leadership positively influenced hygiene behaviors through the mediating effect of organizational factors, and negative traits of leadership negatively influenced HHB through the mediating effect of organizational commitment (Bittner et al., 2002; Ko and Kang, 2019). The results of a study exploring the influences of leadership style and organizational climate on hygiene behaviors showed that leadership style and organizational climate were positively correlated with employees’ hygiene behaviors and the organizational climate had a complete mediating effect (Ko and Kang, 2019). A study of an intervention involving visual performance feedback reported that leadership and organizational commitment negatively impacted HHB because of low leadership support and organizational commitment (Bittner et al., 2002).

To support further promotion of HHB among medical staff, and based on the research status that there were no studies on the impact mechanism of the three variables. Our study aimed to fill the gap by exploring the impacts of self-expectation leadership and organizational commitment on the HHB of medical staff based on the ILT. According to the theoretical framework and literature review, the following hypotheses were examined in this study.

Hypothesis 1: Positive traits of self-expectation leadership had a positive effect on organizational commitment.

Hypothesis 2: Negative traits of self-expectation leadership had a negative effect on organizational commitment.

Hypothesis 3: Organizational commitment had a positive effect on HHB.

Hypothesis 4: Positive traits of self-expectation leadership had a positive effect on HHB.

Hypothesis 5: Negative traits of self-expectation leadership had a positive effect on HHB.

Hypothesis 6: Positive traits of self-expectation leadership positively influenced HHB through the mediating effect of organizational commitment.

Hypothesis 7: Negative traits of self-expectation leadership negatively influenced HHB through the mediating effect of organizational commitment.

## Materials and methods

### Study design, participants, and quality control

A cross-sectional study using an online self-administered and anonymous questionnaire was conducted in all second-level and third-level hospitals in Hubei province (Central China) in May 2022. There were 217 second-level and 90 third-level hospitals, serving 295 million visits in 2021 (NBSC, 2022), and 289 (94.14%) hospitals were surveyed in this study.

Hospital Infection Management Quality Control Center (HIMQCC) sent the questionnaire to the directors of the infection management department in each hospital, and the directors sent the questionnaire to medical staff. The directors were responsible for quality control by checking the questionnaire filling.

Hospital Infection Management Quality Control Center required at least eight medical staff who have time, willingness, and are on duty in the following departments to fill the questionnaire: respiratory, urological, intensive care unit, neurology, endocrinology, and orthopedics, and at least five medical staff in other departments to fill the questionnaire. A total of 23,426 questionnaires were received. The quality control before data analysis was based on the following exclusion criteria, and 21,917 valid questionnaires were obtained with an effective response rate of 93.56%.

1. The unreasonable answer, e.g., too long clinical work year.
2. Short answer time. The time required to answer the questionnaire was not less than 10 min (The minimum answer time tested by our research group was 12 min).
3. Inconsistent answers to trap items. Our research group set up two items with the same but different expressions in the questionnaire (trap items). If the answers to the two items were inconsistent, we excluded the questionnaire.

### Questionnaire’s measurement and modification

The theoretical framework of this study is required to assess positive and negative traits of self-expectation leadership, organizational commitment, and HHB. All of the assessment tools have been evidenced suitable to apply in the healthcare field (Chang and Chang, 2009; Alabdulhadi et al., 2017; Chen et al., 2017). Besides, staff demographics, such as age and professional title, were also collected. The specific measurements were presented as follows.

Medical staff’s positive and negative traits of self-expectation leadership were measured based on Offermann’s eight dimensions scale, including sensitivity, dedication, tyranny, charisma, attractiveness, masculinity, intelligence, and strength (Offermann et al., 1994). According to the Chinese cultural situation and the latest development of Offermann’s scale, we also added moral and creative dimensions to measure self-expectation leadership (Ling and Chen, 1987; Offermann and Coats, 2018). At the same time, combined with the background of HAI prevention and control, the attractiveness dimension was deleted, and similar items were combined such as combining “considerate of others” and “sympathetic of others,” a total of 18 items were obtained. The positive and negative traits of self-expectation leadership reliability with Cronbach’s coefficient alpha values were 0.87 (before modification), 0.98 (after modification), 0.82 (before modification), and 0.966 (after modification). The confirmatory factor analysis (CFA) was performed to assess the factor structure, demonstrating good construct validity (the factor loadings were not less than 0.5, and after modification were not less than 0.9). The items were responded to with a 10-point scale, 1 referred to totally inconformity and 10 referred to totally conformity. Based on the background of HAI prevention and control, higher scores of positive traits of self-expectation leadership indicated a greater leadership level, while higher scores of negative traits of self-expectation leadership indicated a lesser leadership level in this study.

Organizational commitment was measured by Chang’s three dimensions scale, including value commitment, effort commitment, and retention commitment (Chang and Chang, 2009; Chen et al., 2017). After modifying the statement of the scale to adapt the survey unit (department) in this study, such as modifying “I pay attention to the future development of the hospital” to “I pay attention to the future development of the department,” a total of 12 items were obtained. The reliability with Cronbach’s coefficient alpha values was 0.862 (before modification) and 0.975 (after modification), and the factor loadings of CFA were not less than 0.4 (before modification) and 0.8 (after modification). The items were responded to on a Likert 5 scale, 1 referred to strongly disagree and 5 referred to strongly agree. Higher scores indicated a greater organizational commitment level.

The HHB was measured by a self-designed scale. Chinese *specification of hand hygiene for healthcare workers* was used to design the scale (National Health Commission of the People’s Republic of China, 2019; Li and Xu, 2020), and a total of 12 items were obtained. The reliability with Cronbach’s coefficient alpha values was 0.975, and the factor loadings of CFA were not less than 0.9. The items were also responded to on a Likert 5 scale, 1 referred to strongly disagree and 5 referred to strongly agree. Higher scores indicated a greater HHB level.

The questionnaire was tested by an HAI infection prevention and control professor in a third-level hospital and a sample of 15 experts in a teaching university to improve the internal validity. They were asked to complete the questionnaire and provide verbal feedback regarding the items’ readability. Some items were reworded according to the verbal feedback.

### Statistical analysis

In this study, IBM SPSS Statistics version 25.0 and Amos 28.0 were jointly used to conduct the statistical analyses.

The structural equation model (SEM) was applied to explore the relationship among medical staff’s positive and negative traits of self-expectation leadership, organizational commitment, and HHB based on the theoretical framework (HHB model) as SEM can simultaneously test the factor structure of latent variables and the complex relationships among multiple variables, such as direct and indirect relationships (MacKinnon et al., 2002). Since the responses of each item were 5 or 10-point scale (ordinal variables), means and variance adjusted weighted least squares extraction estimation was applied to examine the associations among the study variables.

The goodness of fit indices were applied to evaluate the fit of the structural equation model: Root mean square error of approximation (RMSEA; <0.08 acceptable, <0.05 excellent), normed fit index (NFI; >0.90 excellent), incremental fit index (IFI, >0.90 excellent), comparative fit index (CFI; >0.90 acceptable, >0.95 excellent), and parsimony-adjusted GFI (PGFI; >0.50 excellent) (Bentler, 1990; Hu and Bentler, 1999).

## Results

### Demographics description

Most of the medical staff surveyed were female subjects (81.50%). Their average age was 33.800. Nearly 90% of staff had a university and above degree, and over 60% of them worked in third-level hospitals. Nearly 70% of them were nurses, and the staff’s average clinical working year was 11.140. Most of them held middle or primary job titles (84.50%), equally distributed in all departments. The demographics of the medical staff are shown in Table 1.

**TABLE 1:** Demographics of medical staff (*n* = 21917).

Demographic	*N* (%)/Mean ± SD
**Gender**	
Male	4,068 (18.50)
Female	17,903 (81.50)
Age	33.800 ± 7.545
**Educational level**	
Junior college and below	2,801 (12.70)
University degree	15,765 (71.80)
Master degree and above	3,405 (15.50)
**Facility setting**	
Second-level hospital	7,971 (36.30)
Third-level hospital	14,000 (63.70)
**Occupation**	
Physician	6,723 (30.60)
Nurse	15,248 (69.40)
Clinical working year	11.140 ± 8.004
**Professional title**	
No title	881 (4.00)
Primary title	10,278 (46.80)
Middle title	8,286 (37.70)
Vice-senior title	2,090 (9.50)
Senior title	436 (2.00)
**Department**	
Respiratory Department	1,851 (8.40)
Urological Department	1,143 (5.20)
Intensive Care Unit	1,660 (7.60)
Neurology Department	1,968 (9.00)
Endocrinology Department	1,184 (5.40)
Orthopedics Department	2,209 (10.10)
Internal Medicine	3,901 (17.80)
Surgery Department	3,312 (15.10)
Pediatrics Department	2,316 (10.50)
Obstetrics and Gynecology Department	2,427 (11.00)

### Measurement score and correlation analysis of medical staff’s self-expectation leadership, organizational commitment, and hand hygiene behavior

As shown in Table 2, the measure score of positive traits of self-expectation leadership was 9.115 ± 1.391, negative traits of self-expectation leadership were 3.615 ± 3.210, an organizational commitment was 4.484 ± 0.693, and HHB was 4.792 ± 0.444. Correlation analysis showed that positive traits of self-expectation leadership were significantly positively correlated with organizational commitment and HHB (*p* < 0.01), while negatively correlated with negative traits of self-expectation leadership (*p* < 0.01). Negative traits of self-expectation leadership were significantly negatively correlated with organizational commitment, while positively correlated with HHB (*p* < 0.01). Organizational commitment was significantly positively correlated with HHB (*p* < 0.01).

**TABLE 2:** Measurement score and correlation analysis of self-expectation leadership, organizational commitment, and hand hygiene behavior.

Variable	Mean ± SD	Correlation coefficient			
		1	2	3	4
(1) Positive traits of self-expectation leadership	9.115 ± 1.391	1			
(2) Negative traits of self-expectation leadership	3.615 ± 3.210	–0.228**	1		
(3) Organizational commitment	4.484 ± 0.693	0.623**	–0.156**	1	
(4) Hand hygiene behavior	4.792 ± 0.444	0.429**	0.055**	0.517**	1

Positive and negative traits of self-expectation leadership were responded with 10-point, 1 referred to totally inconformity and 10 referred to totally conformity. Organizational commitment and hand hygiene behavior were responded with 5-point, 1 referred to strongly disagree and 5 referred to strongly agree. **p < 0.01.

### Structural equation model for hand hygiene behavior (hand hygiene behavior model)

**FIGURE 1:**
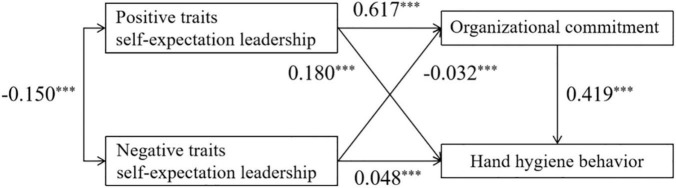
The results of hand hygiene behavior (HHB) model based on the implicit leadership theory (ILT) theoretical framework. ^***^p < 0.001. Model fit indices: RMSEA = 0.076; NFI = 0.903; IFI = 0.903; CFI = 0.903; PGFI = 0.644.

The hand hygiene behavior model based on the ILT theory showed good model fit indices with RMSEA = 0.076 (acceptable), NFI = 0.903 (excellent), IFI = 0.903 (excellent), CFI = 0.903 (excellent), PGFI = 0.644 (excellent), and the detail information is shown in Figure 1. Positive and negative traits of self-expectation leadership were identified as significant predictors of organizational commitment and medical staff’s HHB, and organizational commitment was also demonstrated as one significant predictor of HHB.

As shown in Table 3, positive traits of self-expectation leadership had a positive effect on organizational commitment (β = 0.617, *p* < 0.001) and HHB (β = 0.180, *p* < 0.001). Negative traits of self-expectation leadership had a negative effect on organizational commitment (β = –0.032, *p* < 0.001), while a positive effect on HHB (β = 0.048, *p* < 0.001). The organizational commitment had a positive effect on HHB (β = 0.419, *p* < 0.001). The mediating effect of the organizational commitment showed positively between positive traits of self-expectation leadership and HHB (β = 0.259, *p* < 0.001), while negatively between negative traits of self-expectation leadership and HHB (β = -0.013, *p* < 0.001).

**TABLE 3:** Direct, indirect, and mediating effects of self-expectation leadership, organizational commitment, and hand hygiene behavior.

Path	β	SE	*P*	Percentile 95% CI	
				Lower	Upper
A → C → D	0.259	0.008	<0.001	0.246	0.272
A → C	0.617	0.003	<0.001	0.605	0.629
C → D*	0.419	0.245	<0.001	0.399	0.439
A → D	0.180	0.057	<0.001	0.158	0.202
B → C → D	–0.013	0.002	<0.001	–0.017	–0.010
B → C	–0.032	0.001	<0.001	–0.041	–0.023
B → D	0.048	0.006	<0.001	0.040	0.056

A referred to the positive trait of self-expectation leadership, B referred to the negative trait of self-expectation leadership, C referred to organizational commitment, and D referred to hand hygiene behavior; A → C → D and B → C → D were mediating effects, A → D and B → D were direct effects, others were indirect effects. *The indirect effects of C → D were the same between A → C → D and B → C → D.

## Discussion

This is the first study to explore the impacts of self-expectation leadership and organizational commitment on the HHB of medical staff based on the ILT. Our study revealed that positive and negative traits of self-expectation leadership were significant predictors of organizational commitment and medical staff’s HHB, and organizational commitment was also demonstrated as one significant predictor of HHB. Additionally, there were positively mediating the effect of the organizational commitment between positive traits of self-expectation leadership and HHB, while negatively mediating the effect between negative traits of self-expectation leadership and HHB. However, the medical staff’s negative traits of self-expectation leadership showed a low path coefficient of direct, indirect, and mediating effects on organizational commitment and HHB.

Consistent with the results of Chen’s, Sangperm’s, Lieber’s, and Teoh’s studies that positive traits of self-expectation leadership were positively associated with organizational commitment and HHB (Chen, 2011; Lieber et al., 2014; Sangperm, 2017; Teoh et al., 2022), our study demonstrated the same results in the field of HAI prevention and control. Research from the field of psychology explained the possible reason for the results that medical staff with high positive traits of self-expectation leadership were more active and committed in the organization and displayed creativity and initiative in their work behavior (DiLiello and Houghton, 2006).

Congruent with previous studies, negative traits of self-expectation leadership were negatively associated with organizational commitment but positively associated with HHB (Chen, 2011; Pereira and Stornelli, 2022). However, it was worth noting that negative traits of self-expectation leadership had a limited effect on the two variables in this study. The main reasons lay in the characteristics of negative traits of leadership and the increase in emphasis on individual autonomy culture in China (Deng, 2018; Ünler and Kılıç, 2019). Negative trait leadership also known as paternalistic leadership, emphasizes hierarchical order and leaders’ authority, and it can improve individual behavior levels under a collectivistic cultural background (Cheng et al., 2014). Whereas, in recent years, Chinese residents have increasingly considered their feelings and emphasized themselves in many situations, leading to low behavior compliance (Li and Wu, 2012). Under this contradiction, the results of this study were presented in China.

Consistent with the results of many studies, our study confirmed that organizational commitment was significantly positively associated with HHB (Boscart et al., 2012; Tan and Olivo, 2015). Improving employees’ organizational commitment has been recognized as an important way to improve behavior and performance (Anggraeni and Rahardja, 2018; Luengalongkot et al., 2020). The result of a study from Indonesia showed a significantly positive effect on hand hygiene behavior by organizational commitment (*p* < 0.05) (Sari and Winarno, 2022). In addition, several qualitative studies have reached the same conclusion (Boscart et al., 2012; Tan and Olivo, 2015). A study pointed out the possible mechanism that organizational culture, such as organizational commitment, can improve the behavior and motivation of human resources so as to improve its performance and in turn improve the performance of the organization to achieve organizational goals (Uha, 2013).

There were seldom studies on the mediating effect of organizational commitment between positive or negative traits of self-expectation leadership and HHB. But in the similar field of healthcare, some studies were speculated to show the possible relationship between the variables that organizational commitment played a positive role between positive traits of self-expectation leadership and HHB, a negative role between negative traits of self-expectation leadership and HHB (Bittner et al., 2002; Ko and Kang, 2019). The organizational commitment had a relatively high influence between positive traits of self-expectation leadership and HHB and a low influence between negative traits of self-expectation leadership and HHB. Positive traits of self-expectation leadership were clearly evidenced to improve employees’ perceptions, attitudes, and then behavior (Kolzow, 2014). Although negative traits of leadership have been confirmed that can improve employees’ behavior levels by forcing them, such coercive measures can easily bring dissatisfaction, which will lead to limited and unsustainable behavior improvement (verywell mind, 2022).

There were also some limitations in this study. First, it relied on medical staff self-report outcomes of the HHB and, thus, may be at risk of social desirability bias (Boivin et al., 2008). Relative to other sources of information, such as direct observation, self-report outcomes were argued to overestimate the true level of HHB (Hoffmann et al., 2020; Gaube et al., 2021). Besides, questionnaires administered by IPC professionals may also lead to an unreal level of HHB. Second, this study was conducted in a second and third-level hospitals in Hubei province, there were between and within cluster effects, as medical staff in the same hospitals may tend to respond similarly, in different hospitals may tend to respond differently. Thus, the results should be interpreted with caution.

## Conclusion

In this study, SEM and ILT were used to explore the impacts of self-expectation leadership and organizational commitment on the HHB of medical staff. The main study findings showed that positive traits of self-expectation leadership had a positive effect on organizational commitment and HHB. Negative traits of self-expectation leadership had a limited negative effect on organizational commitment, while a limited positive effect on HHB. The organizational commitment had a positive effect on HHB. The mediating effect of organizational commitment showed positively between positive traits of self-expectation leadership and HHB, while limited negatively between negative traits of self-expectation leadership and HHB. It suggests that positive traits of self-expectation leadership are important predictors of promoting organizational commitment and HHB, while negative traits of self-expectation leadership have a limited impact on organizational commitment and HHB in the field of HAI prevention and control. This study, on the one hand, can further develop the theory of implicit leadership (expand the theory into positive and negative traits of self-expectation leadership) and apply the theory in the field of HAI prevention and control. On the other hand, different leadership traits have also been found different effects on organizational commitment and HHB, suggesting the need to focus on positive traits of self-expectation leadership, although negative traits of self-expectation leadership can also promote HHB to a lesser degree among medical staff, it will reduce their organizational commitment.

## Data availability statement

The raw data supporting the conclusions of this article will be made available by the authors, without undue reservation.

## Ethics statement

This study was approved by the Ethics Committee of Tongji Medical College, Huazhong University of Science and Technology (2021-S063). Written informed consent was not required for participation in accordance with national legislation.

## Author contributions

QW, XL, XZ, QZ, and LT conducted the study conceptualization and design. QW, FZ, TY, LW, YW, and KW carried out the data collection. QW conducted the data analysis and original draft preparation. XZ, QZ, and LT reviewed and edited the manuscript. All authors contributed to the article and approved the submitted version.

## Abbreviations

HHB: hand hygiene behavior
HAI: healthcare-associated infection
IPC: infection prevention and control
ILT: implicit leadership theory
HIMQCC: hospital infection management quality control center
CFA: confirmatory factor analysis
SEM: structural equation model
RMSEA: root mean square error of approximation
NFI: normed fit index
IFI: incremental fit index
CFI: comparative fit index
PGFI: parsimony-adjusted GFI
HHB model: structural equation model for hand hygiene behavior
SD: standard deviation
SE: standard error
95% CI: 95% confidence interval.
